# A spatial and temporal analysis of notifiable gastrointestinal illness in the Northwest Territories, Canada, 1991-2008

**DOI:** 10.1186/1476-072X-11-17

**Published:** 2012-05-29

**Authors:** Aliya Pardhan-Ali, Olaf Berke, Jeff Wilson, Victoria L Edge, Chris Furgal, Richard Reid-Smith, Maria Santos, Scott A McEwen

**Affiliations:** 1 Department of Population Medicine, University of Guelph, Guelph, ON, Canada; 2 Department of Mathematics and Statistics, University of Guelph, Guelph, ON, Canada; 3 Novometrix Research Inc, Moffat, ON, Canada; 4 Department of Indigenous Environmental Studies, Trent University, Peterborough, ON, Canada; 5 Department of Health and Social Services, Government of the Northwest Territories, Yellowknife, NT, Canada

**Keywords:** Gastrointestinal illness, Foodborne diseases, Waterborne diseases, Minority health, Population surveillance, Spatial epidemiology, Temporal epidemiology, Spatio-temporal epidemiology

## Abstract

**Background:**

This is the first study to describe the geographical and temporal distribution of notifiable gastrointestinal illness (NGI) in the Northwest Territories (NWT), Canada. Understanding the distribution of NGI in space and time is important for identifying communities at high risk. Using data derived from the Northwest Territories Communicable Disease Registry (NWT CDR), a number of spatial and temporal techniques were used to explore and analyze NGI incidence from the years 1991 to 2008. Relative risk mapping was used to investigate the variation of disease risk. Scan test statistics were applied to conduct cluster identification in space, time and space-time. Seasonal decomposition of the time series was used to assess seasonal variation and trends in the data.

**Results:**

There was geographic variability in the rates of NGI with higher notifications in the south compared to the north. Incidence of NGI exhibited seasonality with peaks in the fall months for most years. Two possible outbreaks were detected in the fall of 1995 and 2001, of which one coincided with a previously recognized outbreak. Overall, incidence of NGI fluctuated from 1991 to 2001 followed by a tendency for rates to decrease from 2002 to 2008.

**Conclusions:**

The distribution of NGI notifications varied widely according to geographic region, season and year. While the analyses highlighted a possible bias in the surveillance data, this information is beneficial for generating hypotheses about risk factors for infection.

## Background

Notifiable gastrointestinal illness (NGI) constitutes a major cause of morbidity and mortality worldwide. In developed countries, mortality from NGI is infrequent but illness is common and the socio-economic burden is reported to be high [1]. Enteric pathogens are often transmitted to humans via food or water and result in diarrhea or vomiting as well as fever, cramps, nausea and headache [2]. The time between exposure and the manifestation of symptoms can vary from a few hours to weeks, depending on the agent [3]. To date, numerous studies have described spatial and temporal patterns of NGI within areas of Canada and other countries; however, NGI in the Northwest Territories (NWT), is a largely under-studied area in the literature [4-7].

**Figure 1:**
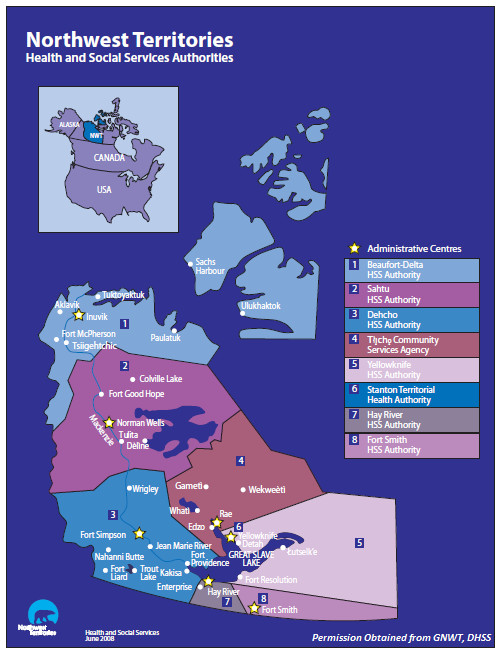
Map of the NWT showing locations of communities and Health and Social Services Authorities.

The NWT is a federal territory located in Northern Canada and is comprised of 33 communities (Figure 1); of these, most are in remote locations with fewer than 1,000 residents. Behchoko, Fort Smith, Hay River, Inuvik, and Yellowknife are the only communities that have a population greater than 2,000 [8]. Many rural/remote communities are only accessible by air or, during winter months, by ice roads [9].

According to the 2006 census, the NWT has a population of 41,464 with Aboriginal people representing the majority (50.3%). About 61% (12,640) of all Aboriginal people in the territory are First Nations while 20% (4,200) are Inuit and 17% (3,600) are Métis. Yellowknife has the largest number of Aboriginal (First Nations, Métis and Inuit) residents, 4,105 (22.2%). Behchoko (formerly known as Rae-Edzo) has the largest First Nations community, 1,730 (91.5%) while Inuvik has the largest Inuit population, 1,335 (38.9%) [10].

The Aboriginal populations of the NWT maintain a strong connection with the environment through harvesting, hunting, fishing and trapping [11]. Animal and plant species acquired through these activities, also referred to as traditional or country foods, provide significant nutritional value and the sharing of these foods strengthens social ties and promotes cultural exchanges [12]. Studies of outbreaks in Aboriginal communities however, have indicated some of the pathways of exposure to agents of food and waterborne illness through these activities [13-17]. Bacteria, viruses, parasites or fungi may be present on skin or fur, within the gastrointestinal tract, and in the various tissues, organs and meat of wild game and fish or in untreated surface water [8,18]. Food and water safety have become major concerns in northern populations, particularly the increased risk of exposure to pathogens such as *E. coli, Salmonella* and *Campylobacter* through the preparation, storage and consumption of wild game, as well as infection from *Giardia* and *Cryptosporidium* from the consumption of untreated surface water or marine mammals which act as reservoirs for these diseases [11,19-23].

Since 1988, the Government of the NWT (GNWT) has assumed full control of health services with responsibilities shared between eight regional Health and Social Services Authorities (HSSAs) (Figure 1) [24]. Under the guidelines of the Canada Health Act, the GNWT ensures that all residents enrolled in the NWT Health Care Plan (also called Insured Health Benefits) have access to medically necessary physician and hospital services with no payment required. They also provide Supplementary Health Benefits such as dental care, prescription drugs, and medical travel for Métis residents, seniors, and people with specific diseases or conditions. Annually, Health Canada provides funding to the GNWT to support Non-Insured Health Benefits for First Nations and Inuit residents [25].

In the NWT, hospitals and physician clinics are located in the communities of Yellowknife, Hay River, Fort Smith and Inuvik. There are primary health care centers across the rest of the territory to serve rural/remote populations, employing between one and seven nurses each. There are no resident general practitioners for these communities; however, there is a visiting service every four to six weeks [26].

At present, there are very little baseline data on NGI in the territory. Moreover, the number of cases of NGI identified through public health surveillance systems is recognized to be a significant underestimate of the true burden of illness [27]. Under-reporting may be further exacerbated in rural/remote areas of the NWT, particularly due to ongoing challenges with maintaining and supporting human resources, health infrastructure, and long-distance travel to facilities [28]. Given their geographic location, socio-cultural practices and preferences, rural/remote communities of the NWT are potentially at greater risk for acquiring infections related to pathogenic agents in the environment. The entire population of the NWT may also be exposed to enteric hazards in: contaminated non-traditional retail foods; contaminated traditional foods commonly found frozen in grocery stores, prepared in specialty restaurants and purchased online; and through the consumption or recreational use of contaminated water. Given this unique context, there is a genuine need to identify populations at high risk for disease and generate hypotheses about potential risk factors that may differ by area [7,29-31]. This information is useful for guiding individual-level studies and for aiding health authorities in strategically directing preventative health care resource programs to areas of greatest need [30,32]. Therefore, the objective of this study was to investigate temporal and geographical distributions of NGI using data extracted from the Northwest Territories Communicable Disease Registry (NWT CDR) from 1991 to 2008.

## Results

### Purely spatial cluster investigation

**Figure 2:**
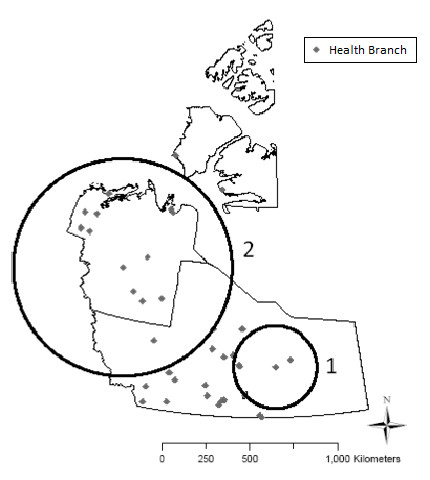
Significant (p ≤ 0.05) spatial clusters of notifiable gastrointestinal illness in the NWT, detected using a scan test.

Applying a spatial scan test using a scanning window size of up to 50% of the population at risk, two significant spatial clusters were detected, one consisting of high rates and the other with low rates (Table 1 and Figure 2). The primary cluster of high rates, Cluster 1, was centered in the Yellowknife HSSA and had a relative risk (RR) of 1.53 (p ≤ 0.01). The primary cluster of low rates, Cluster 2 (RR = 0.62, p ≤ 0.01) was located in the Sahtu and Beaufort Delta HSSAs.

**Table 1:** Significant (p ≤ 0.05) spatial clusters of notifiable gastrointestinal illness in the NWT, detected using a scan test

	**Location (HSSA)**	**Population**	**Cases**	**p-value**	**Relative risk**	**Radius (km)**
**Cluster 1**	Yellowknife	20,598	413	0.001	1.53	218.49
**Cluster 2**	Beaufort Delta & Sahtu	7,980	88	0.001	0.62	465.15

### Purely temporal clusters

With the temporal scanning window set to a maximum of up to 50% of the study period, two significant temporal clusters were detected; one high rate and one low rate cluster (Table 2). The primary cluster of high rates, Cluster 1 (RR = 4.25, p ≤ 0.01) occurred during October 2001 to November 2001. The primary cluster of low rates, Cluster 2 (RR = 0.56, p ≤ 0.01) occurred from November 2002 to May 2008.

**Table 2:** Significant (p ≤ 0.05) temporal clusters of notifiable gastrointestinal illness in the NWT, detected using a scan test

	**Location (HSSA)**	**Cases**	**p-value**	**Relative risk**	**Time** **frame**
**Cluster 1**	All	27	0.001	4.25	2001/10-2001/11
**Cluster 2**	All	142	0.001	0.56	2002/11-2008/5

### Spatio-temporal clusters

**Figure 3:**
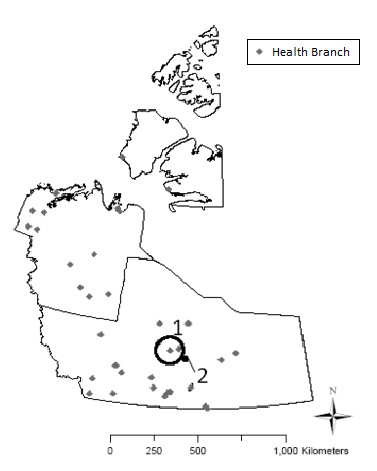
Significant (p ≤ 0.05) spatio-temporal clusters of notifiable gastrointestinal illness in the NWT, detected using a scan test.

The NWT CDR does not indicate whether cases of NGI are associated with an outbreak. The details of two outbreak investigations have been published in EpiNorth newsletters including an outbreak of salmonellosis in October 1995 in Yellowknife [33] and an outbreak of botulism in August 1997 in Arviat (a community in the NWT prior to the establishment of the Nunavut Territory in 1999) [34]. We explored the occurrence of new or previously identified outbreaks as clusters of NGI during the study period. With a scan window of 60 days and 50% of the population at risk, the spatio-temporal scan found two clusters of high rates and no clusters of low rates (Table 3 and Figure 3). The primary cluster of high rates, Cluster 1 (RR = 37.25, p ≤ 0.01) occurred between October 2001 to November 2001 in Behchoko and Whati (Tlicho Community HSSA). A secondary cluster of high rates, Cluster 2 (RR = 10.12, p ≤ 0.01) occurred during the month of October 1995 in Yellowknife (Yellowknife HSSA) which coincides with a previously identified outbreak.

**Table 3:** Significant (p ≤ 0.05) spatio-temporal clusters of notifiable gastrointestinal illness in the NWT, detected using a scan test

	**Location (HSSA)**	**Population**	**Cases**	**p- value**	**Relative risk**	**Radius (km)**	**Time frame**
**Cluster 1**	Behchoko & Whati	2,515	14	0.001	37.25	73.57	2001/10- 2001/11
**Cluster 2**	Yellowknife	19,506	15	0.001	10.12	8.29	1995/10

### Relative risk map

**Figure 4:**
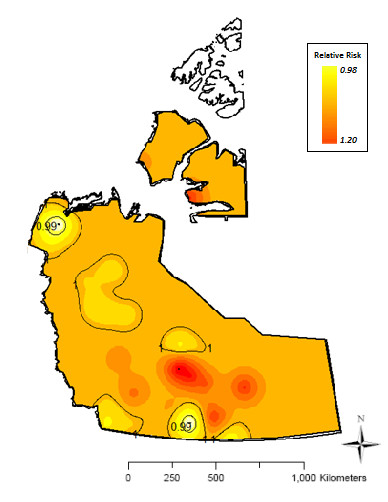
Relative risk Map of notifiable gastrointestinal illness in the NWT.

The spatial relative risk function for NGI is mapped in Figure 4. The color scale ranged from shades of red to yellow indicating high, to low risk, respectively. Isolines on the map depicted levels of equal risk in probabilities per square kilometer. The raw risk varied from 0 to 2.8%. The smooth risk varied between 1.2% and 2.0%. The smoothed background risk was estimated at 1.57%. The relative risk surface showed no relevant spatial variation, with predicted relative risks ranging between 0.98 and 1.20.

### Time series and seasonal decomposition

**Figure 5:**
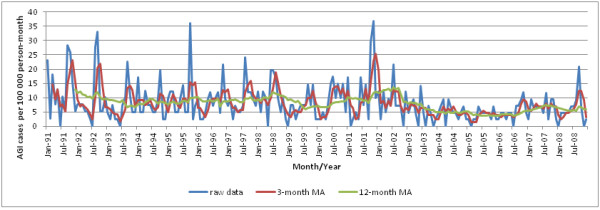
Time series of monthly incidence of notifiable gastrointestinal illness in the NWT, with moving averages (MA).

**Figure 6:**
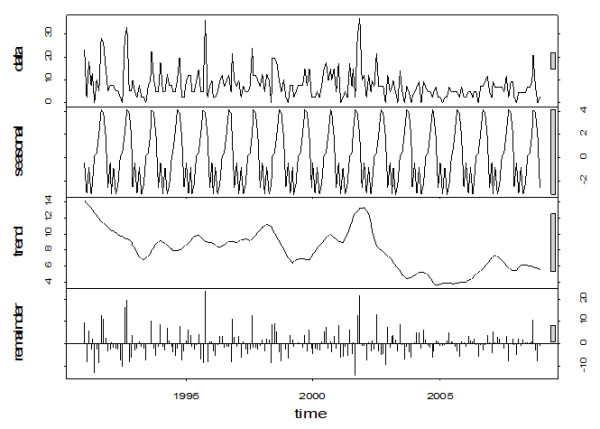
Seasonal decomposition of monthly incidence of notifiable gastrointestinal illness in the NWT.

Time series and seasonal decomposition plots can be seen in Figures 5 and 6, respectively. Visual inspection of the plot suggests that disease incidence decreased from 1991 to 1993, followed by an increase in 1994. Incidence remained relatively stable from 1995 to 1998. In 1999, disease incidence decreased again followed by an increase from 2000 to mid-2002. In mid-2002, the trend decreased dramatically to a 50% lower level for the rest of the study period. Overall, the incidence rate of NGI had a significant negative trend (p ≤ 0.01), indicating that the incidence was decreasing over the study period. Seasonal decomposition of the incidence indicated peak occurrence in the fall months (September, October and November) with smaller secondary peaks in the spring (March, April and May) until 2002. After 2002, seasonality was not easily identifiable but several strong peaks appeared in the summer months (June, July and August).

## Discussion

The results from the analyses highlighted three major patterns in the incidence of NGI in the NWT from 1991 to 2008: differences in the localities of high versus low spatial clusters in the study area; a decrease in reported incidence over the last five years of the study period; and marked seasonality in fall and spring months. Due to the paucity of case information, particularly over a large geographic area (an inherent problem of northern rural/remote communities), the results should be interpreted with some degree of caution.

Based on the scan test, the two spatial clusters revealed higher-than-expected incidence of NGI in the southern portion of the NWT and lower-than-expected rates in the north. These cluster patterns may reflect true differences in risk or may be due to under-reporting and/or under-diagnosis in the north. Due to low population densities and small patient volumes in rural/remote areas, community health facilities focus on providing primary care and emergency care locally which may lead to under-diagnosis of uncomplicated cases of NGI. Alternatively, rural/remote patients may rely more extensively on traditional healers or other alternative methods of health care, or they may forego treatment altogether; these cases will not be captured in the existing surveillance system. It is possible however, that there is a truly higher incidence in urban areas which could be due to the geographical distribution of important community-level risk factors/behaviors across the territory. Understanding risk factors is an important element of disease control and therefore, prospective analytical observation studies of risk factors in the NWT, particularly between southern and northern areas, are warranted.

Although the spatial scan test identified areas of high and low risk, there was no relevant spatial variation in predicted relative risk. The differences in raw risk among communities are likely an effect of varying population at risk. Due to the sparse spatial sample size and large distances between centroids of administrative regions, spatial correlation could not be identified from raw data, and thus regional variation was averaged out across the territory resulting in a spatially flat map [35,36].

The purely temporal cluster of low rates which occurred from the end of 2002 through mid-2008 is an indication that reported NGI rates in the NWT, regardless of spatial location, have decreased over the last few years of the study period. Visual inspection of the time series also suggested a downward trend beginning in mid-2002. Several programs such as enhanced surveillance, better provision of drinking water and health promotion programs may have contributed to these changes in some areas [37]. Alternatively, changes in case criteria, diagnostic procedures, reporting practices and population demographics may have contributed to lower rates across the territory [38]. The extent to which each of these factors may have contributed to a decrease in incidence is unknown and beyond the scope of this study but it is an important topic for future research.

Two spatio-temporal clusters of high rates of NGI were also detected in the NWT. In October 1995, a large cluster was detected in Yellowknife. This cluster coincided with a known salmonellosis outbreak in Yellowknife attributed to the consumption of undercooked pork at a Thanksgiving Pig Roast held on October 7, 1995 [33,39]. From October through November 2001, another cluster was detected in the Tlicho HSSA. Further investigation into the NWT CDR revealed that these were all cases of cryptosporidiosis. This outbreak was traced back to an asymptomatic carrier of cryptosporidiosis who originated from outside the NWT.

Seasonal decomposition of the time series revealed significant seasonality in spring and fall months for most years. Several studies in Southern Canada have shown similar seasonal patterns of NGI [40-42]. In the NWT, seasonal peaks over the study period may have been attributed to environmental and social factors such as higher ambient temperatures, frequent travel for subsistence activities, centralized outdoor meal preparation as well as the consumption of animal foods and surface water. Additional seasonal peaks during summer months after 2002 appear to be associated temporally with warming trends. Numerous studies forecast that long-term warming due to climate change will alter the population size, length of transmission season as well as the range of hosts and pathogens in the north [21,43,44]. Environment Canada has found that over the last 15 years, temperatures in the NWT have been warmer in all seasons with increased (and highly variable) precipitation [45]. It is evident that global warming has already triggered weather changes in the NWT but the statistically significant decreasing trend of NGI incidence suggests that predicted temperature-driven increase of enteric diseases has not yet begun. The spread of disease, however, depends on a much broader range of ecological and societal factors; further investigation is warranted.

## Conclusions

Disease mapping highlighted the spatial distribution of high and low notification rates in the NWT and these were confirmed by cluster analyses. The results of the spatial analyses indicate that higher rates of NGI were observed in urban areas of southern NWT which may be due to a true higher incidence, differential reporting and/or the geographical distribution of risk factors. Overall, there was a significant decrease in reported rates over time; if real, it is unclear if these rates have in fact been decreasing or that this is simply an artifact of changes in the health care system, such as testing or reporting. Therefore, factors such as accuracy, completeness, timeliness, and non-random reporting must be considered when analyzing and interpreting surveillance data. In addition, seasonal variation from year to year suggests that timing of infection may be closely related to environmental or behavioral variables. The use of statistical techniques to correlate health data with socio-economic, cultural and environmental variables such as weather, would allow the impact of these factors on human health to be examined and better understood in a northern context [29,31]. By applying various spatial and temporal analytical techniques, we were able examine areas with high (or low) disease rates, identify potential outbreaks, determine seasonality and trends in disease patterns as well as generate hypotheses about risk factors of infection for future studies which can play a key role in prevention and control.

## Methods

### Case data

Data on 708 notifications of reported NGI were obtained from the NWT CDR for the time period January 1, 1991 to December 31, 2008. Reported NGI is an umbrella term for 15 enteric, food- and waterborne conditions that were notifiable under the NWT Public Health Act during the study period: amoebiasis, botulism, brucellosis, campylobacteriosis, cryptosporidiosis, infection with *Escherichia coli*, food poisoning, giardiasis, hepatitis A, listeriosis, salmonellosis, shigellosis, tapeworm, tularemia, and yersiniosis inclusive. The three largest contributors to the total number of notifications were giardiasis with 205 cases (29.0%), salmonellosis with 202 cases (28.5%) and campylobacteriosis with 175 cases (24.7%). Very few cases were attributed to other agents (<6% each). Small case counts over the study period caused a sparse data problem both for detecting cluster patterns with high spatial variability and for discussing results in a way that preserves confidentiality in small communities. Therefore, the focus of our analysis was on NGI rather than individual pathogens. While there are indeed differences among them in terms of agent characteristics, they may share environmental exposure factors (e.g. water, food).

Each case record included details of age, gender, report date, community (place of residence), health branch (place of treatment), disease, etiologic agent, subtype, and suspected exposures. Health branch rather than community was considered to be a better indicator of the place of likely exposure, as individuals tend to move frequently within the territory without changing the permanent address on their health card. Population estimates for health branches from 1991 to 2008 were obtained from the GNWT Bureau of Statistics; therefore, calculations of incidence were based on monthly case counts with denominator re-set annually. The corresponding boundary map was acquired from Statistics Canada. Raw estimates of NGI incidence were geo-located to the centroids of each health branch using spatial join; the point locations of all health branches in the study area were plotted on the NWT map.

### Disease clustering

A set of scan tests [46], implemented in SaTScan software, were used to detect non-random spatial clusters of high (or low) disease rates [47]. The spatial scan statistic is a maximum likelihood ratio test statistic based on a circular window of variable radii scanning the geographical area of interest. The null hypothesis is that disease risk is the same inside as outside the scanning window whereas the alternative hypothesis is that there is elevated (or decreased) risk within the window compared to the outside areas [46]. In our analysis, each reporting health unit was represented by a geographic centroid (longitude and latitude coordinates). At each centroid, a collection of circles of continuously varying radii defined potential spatial clusters; each circle contained its center and neighboring centroids. The radius of the scanning window could be adjusted from 0 to a user-defined *maximum-size*. The *maximum-size* specified the percentage of the maximum total population at risk within the scanning window. Several authors recommended the *maximum-size* to be no greater than 50%; that is, a reported cluster could contain at most 50% of the total population at risk [47-49]. The cluster assessment was carried out through a comparison of the number of cases within the circular window with the number expected if cases were randomly distributed over the at risk population. A Poisson distribution was used to calculate the expected number of cases. A relative risk for each cluster was reported and a p-value was estimated by the Monte Carlo method using 999 replications [47]. Clusters identified at the significance level α = 5% were retained for mapping by polygons around the case locations. Temporal and spatio-temporal scan tests are based on similar procedures with temporal windows covering the entire study area or spatio-temporal cylinders [50]. For this study, the space and time limitations were set to 50% of the study population (to include Yellowknife, which has a population of 18,700) and a minimum of 60 days (to detect potential outbreaks) up to a maximum of 9 years (observe patterns over time).

### Relative risk map

A relative risk map was predicted to give an overview of the risk variation in the study area. A risk map was generated by ordinary kriging of smoothed risk estimates, where smoothing was based on the Empirical Bayes method. Then the risk map was scaled by the background risk. Interpolation is the process of predicting the value of a variable of interest at an unknown location based on known data from neighboring areas which can be used to generate a contour or surface map [51]. Kriging is a statistical method that uses a weighted moving average interpolation for optimal spatial linear prediction. The weights are based on the semivariogram, a function of the distance (and direction) between data locations, and they determine the contribution of each data point to the prediction of new values at unobserved/unsampled locations [51,52]. As is the case in this study, where the population per spatial unit is small with few (or zero) disease events, the area-specific rates are usually unstable (high variances). Consequently, small variations in the number of cases can cause substantial changes in disease risk resulting in maps which are visually dominated by areas with the least stable estimate [53,54]; thus, smoothing of disease rates prior to kriging is recommended. Empirical Bayesian estimation is used to stabilize the variance by “borrowing strength” from all other observations via the global estimate which removes part of the random variation and generates smoothed estimates of risk in each community [53,54].

In this study, the term relative risk has a specific interpretation. All members of the population under study are exposed and at risk; and the risk is the probability of disease occurrence in the population. While the relative risk is generally defined as the risk in the exposed to the unexposed population, another point of view is that part of the population is overexposed (or underexposed) relative to the background exposure which leads to the occurrence of high (and low) rate/risk clusters. The background risk (*r*) is estimated using the results of the spatial scan test using the following equation:

(1) $$r=\left(C–c\right)/\left(N–n\right)\text{,}$$

where *C* and *c* denote the total number of cases and the number of cases in the clusters, respectively. Similarly *N* and *n* denote the size of the population at risk and the size of the population in the disease clusters [54].

### Time series and seasonal decomposition

The time series of monthly NGI incidence rates were plotted together with the smoothed incidence rate series based on monthly and yearly moving averages. A locally weighted regression (Loess) method of seasonal trend decomposition was then used to decompose the time series into a seasonal component, a combined trend and cycle component, as well as an error component [55]. A Seasonal decomposition of monthly incidence rates was then graphed for visual inspection.

### Software

Relative risk mapping and seasonal time series decomposition were carried out in R 2.11.1 (R Development Core Team, New Zealand, 2010). Time series plots and moving average calculations were performed in Excel 2007 (Microsoft Corporation, Washington). Clusters of disease were detected using SaTScan 7.0.2 (Martin Kulldorff and Information Management Services Inc., Massachusetts, 2007).

## Abbreviations

NGI, Notifiable gastrointestinal illness; NWT, Northwest Territories; NWT CDR, Northwest Territories Communicable Disease Registry; GNWT, Government of the Northwest Territories; HSSA, Health and Social Services Authority.

## Competing interests

None

## Authors’ contributions

APA contributed to the manuscript through study design and planning, data collection, analysis and interpretation of results, drafting of manuscript and response to editorial comments and preparation of final manuscript for submission. OB, JW, VLE, CF, RRS and SAM contributed to the manuscript through study design and planning, consultation on study progress, troubleshooting, data analysis and interpretation of results, reviewing and commenting on manuscript drafts. MS contributed to the manuscript through data collection, interpretation of results and reviewing and commenting on manuscript drafts.
